# Prediction of painful temporomandibular joint osteoarthritis in juvenile patients using bone scintigraphy

**DOI:** 10.1002/cre2.175

**Published:** 2019-03-15

**Authors:** Yeon‐Hee Lee, Il Ki Hong, Yang‐Hyun Chun

**Affiliations:** ^1^ Department of Orofacial Pain and Oral Medicine Kyung Hee University Dental Hospital Seoul South Korea; ^2^ Department of Nuclear Medicine Kyung Hee University College of Medicine, Kyung Hee University Hospital Seoul South Korea

**Keywords:** adolescents, bone scintigraphy, cone beam computed tomography (CBCT), osteoarthritis (OA), temporomandibular disorder (TMD)

## Abstract

The study aims to evaluate whether bone scintigraphy is effective in diagnosing temporomandibular joint (TMJ) osteoarthritis (OA) in juvenile patients. A retrospective study was conducted with 356 consecutive patients with TMJ–OA who were clinically assessed according to the Research Diagnostic Criteria for Temporomandibular Disorders. Patients were assigned to three groups based on their ages: Group 1: aged 12–16 years; Group 2: aged 17–19 years; and Group 3: aged 20 years. Additionally, we performed qualitative and quantitative analyses of bone scintigraphy images for the TMJ uptake ratio of the involved joint. The diagnostic rate of TMJ–OA (*n* = 356, 100%), and the overall presence of subjective pain (*n* = 282, 77.3%) was closest to the results of bone scintigraphy (*n* = 333, 91.2%). In addition, reported TMJ pain was significantly associated only with the results of bone scintigraphy and not with the results of panoramic radiography or cone beam computed tomography (CBCT) in all age groups. With CBCT as the reference standard, the optimal cutoff values of the uptake ratio for the diagnosis of TMJ–OA were 2.171 and 2.017 in Groups 1 and 2, respectively (*P* value < 0.05). Our results suggest that bone scintigraphy can be considered a useful modality for diagnosing TMJ–OA in juvenile patients.

## 1 INTRODUCTION

Temporomandibular joint (TMJ) imaging in adolescents is highly challenging because of the small size of the bony components, the possibility of normal variation caused by the growth of a joint, and the lack of clear delineation. The prevalence of temporomandibular disorders (TMDs) in adolescents has been investigated less than it has been in adults but more than it has been in children (Clinical Affairs Committee – Temporomandibular Joint Problems in Children Subcommittee, American Academy of Pediatric Dentistry, 2015). One study revealed that 4.2% of adolescents aged 12–19 years reported TMD pain (Nilsson, 2007), whereas another cross‐sectional study found that 11% of adults aged 20–89 years suffered from TMD pain (Gillborg, Akerman, Lundegren, & Ekberg, 2017). TMJ osteoarthritis (TMJ–OA) is a subtype of TMD, classically defined as degenerative joint disease in the TMJ (Dworkin & LeResche, 1992). Different imaging modalities, including panoramic radiography (PR), cone beam computed tomography (CBCT), and bone scintigraphy, have been used for diagnosing TMJ–OA (Brooks et al., 1997; Epstein, Rea, & Chahal, 2002; Zarb & Carlsson, 1999). The decision to use these diagnostic imaging techniques should be made after considering the age, history and clinical findings, clinical diagnosis, cost of the examination, and amount of radiation exposure, as well as a tentative treatment plan and expected outcome (Brooks et al., 1997). Bone scintigraphy has not been used as a routine diagnostic procedure for TMJ–OA in adolescents because of the potential risk of radiation exposure and the invasiveness of the protocol. Consequently, the usefulness of bone scintigraphy in adolescent TMJ–OA has not been investigated thus far.

**Bullet points**

1. As juvenile TMJ–OA is not an infrequent disease that affects adolescents and is a significant cause of both short‐ and long‐term disabilities, early detection of juvenile TMJ–OA is critical.
2. Our results show that increased uptake in the TMJ region observed on a bone scintigraphy is a relevant, complementary exam, and it can be one of the methods of choice for TMJ–OA diagnosis.
3. Pediatric dentists will be able to prevent juvenile TMJ–OA by noting the signs and symptoms of TMD, as well as the consequence of bone scintigraphy.

Early diagnosis of TMJ–OA is difficult because relatively few symptoms and clinical findings appear at the early stage. In some patients, no signs of TMJ arthritis are seen because TMJ arthritis is not necessarily associated with symptoms or clinical findings (Pedersen, Kuseler, Gelineck, & Herlin, 2008). The Research Diagnostic Criteria for Temporomandibular Disorders (RDC/TMD) is the most widely used diagnostic system for TMDs, including TMJ–OA (Dworkin & LeResche, 1992). Because clinical symptoms and physical examination results are not effective markers of TMJ involvement, imaging plays a crucial role in diagnosis and treatment monitoring. Consequently, the diagnosis of TMJ–OA always includes both clinical examination and imaging procedures of the joint (Muller et al., 2009).

For the early detection of TMJ–OA in adolescents, bone scintigraphy may help to diagnose OA based on increased uptake ratios (Kim et al., 2012). It may be explained with the fact that the sensitivity of bone scintigraphy is considerably high, making it advantageous for the early diagnosis of lesions (Goldstein & Bloom, 1980). Of course, its relatively low specificity is a limitation (Choi et al., 2016; Craemer & Ficara, 1984). An increase in the rate of bone remodeling of approximately 5% is sufficient to cause hyperconcentration of the radioactive compound, whereas PR and CBCT only show bone alteration after 40% to 50% decalcification has occurred (Goldstein & Bloom, 1980). More specifically, bone scintigraphy evaluates the rate of metabolic activity in the skeleton through the use of radiopharmaceuticals, such as Technetium‐99m (^99m^Tc)‐diphosphonate (Coutinho, Fenyo‐Pereira, Dib, & Lima, 2006). Diphosphonates are mainly located in the mineral portion of bone, at active sites of new bone formation and bone resorption, and particularly at the mineral–organic interface at sites of bone remodeling (Brooks et al., 1997; Goldstein & Bloom, 1980). Furthermore, bone scintigraphy reflects the functional change of the TMJ and is valuable in detecting TMJ–OA.

With the increasing use of bone scintigraphy, comprehensive criteria are required for image analysis (Shin et al., 2014) using this nuclear medicine modality, as a part of the RDC/TMD. However, limited information and published data on the juvenile imaging of TMJ–OA have been made available to TMD clinicians. PR is an initial screening tool providing a view of the entire maxillofacial area and not only has the advantage that the radiation exposure is much lower than that of CBCT (Shin et al., 2014; Wrzesien & Olszewski, 2017) but also has a limitation of inadequate for identifying small osseous changes on the surface of the condyle (Epstein, Caldwell, & Black, 2001), particularly in adolescents. The use of CBCT has been accepted as being able to provide an accurate description of TMJ–OA while having a much lower radiation dose than conventional computed tomography (Bag et al., 2014; Zacher, Carl, Swoboda, & Backhaus, 2007). However, the pathologic process can affect growth long before conventional radiographic changes are seen because these radiologic methods are unable to reveal anything more than gross osseous changes. Studying the features of TMJ–OA in adolescent patients using bone scintigraphy is crucial to provide a more comprehensive understanding of its pathophysiologic development and to help in early detection. To our knowledge, the current literature contains no previous investigations evaluating the bone scintigraphy of TMJs in adolescents and comparing them with young adult patients with TMJ–OA. In addition, we also describe the usefulness of this technique by comparing the PR and CBCT imaging techniques.

## 2 PATIENTS AND METHODS

### 2.1 Patients

Among the patients who visited Kyung Hee University Dental Hospital from January 2013 to July 2017, 356 patients (712 TMJs, 143 males and 213 females) aged between 12 and 20 years (mean age: 17.37 ± 2.51 years) and clinically diagnosed with TMJ–OA were included in the study. They were assessed according to RDC/TMD Axis I (Dworkin & LeResche, 1992) and underwent complete radiological examination, including PR, CBCT, and bone scintigraphy. These three imaging techniques were used to diagnose TMJ–OA; the results of which were interpreted by two experts who were blinded to clinical information.

The clinical assessments included mandibular range of motion, the presence of TMJ pain on palpation and on jaw functions, and the presence of TMJ noise. The presence of self‐reported TMD pain was investigated, and its average pain intensity level was rated on a visual analog scale. Exclusion criteria included jaw trauma, mandibular fracture, a history of TMJ surgery, and polyarthritis, as well as patients with missing data.

If arthritis begins before the age of 16 with an unknown etiology, juvenile idiopathic arthritis can be an initial diagnosis (Engstrom, Wanman, Johansson, Keshishian, & Forsberg, 2007; Petty et al., 2004). In addition, skeletal and physical development and growth rates vary according to the different stages of adolescence (Ruff, 2003). Those in their early, middle, and late teens who are growing and those in their 20s who have completed their growth may differ in pain experience/behavior in many ways. Thus, adolescents under the age of 16 years and adolescents between the ages of 17 and 19 years were divided into separate groups, and the results were also compared with those of 20‐year‐old adults. That is, the patients who fulfilled the inclusion criteria were divided, according to their age, into Group 1 (adolescents aged 12–16 years), Group 2 (adolescents aged 17–19 years), and Group 3 (young adults aged 20 years).

This retrospective study was conducted in accordance with the Declaration of Helsinki and was approved by the institutional review board of our institute. Informed consent was obtained from all patients.

### 2.2 The acquisition and analysis of PR and CBCT images

**Figure 1:**
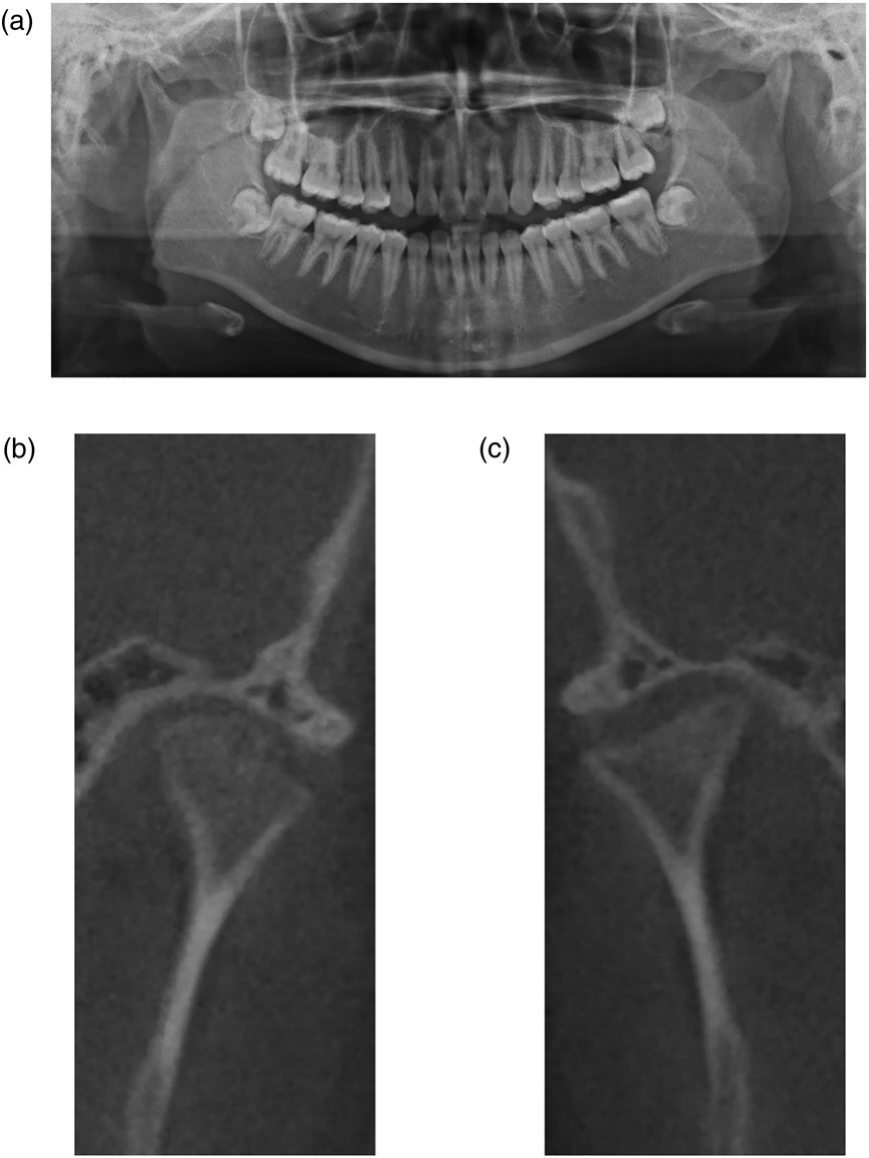
Visual diagnosis of temporomandibular joint osteoarthritis with panoramic radiography (PR) and cone beam computed tomography (CBCT) images. (a) A representative PR image of a 14‐year‐old female with temporomandibular disorder symptoms. A coronal CBCT for the same patient showed erosive bone change, sclerosis, flattening on the right condyle (b), and surface irregularity of the left condyle (c)

**Figure 2:**
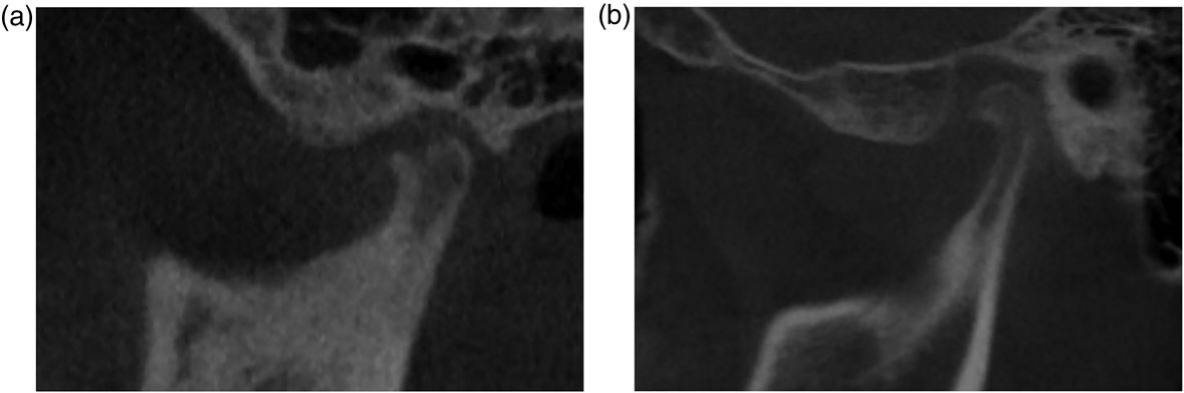
Representative sagittal cone beam computed tomography (CBCT) images showing temporomandibular joint osteoarthritis status. (a) A representative sagittal CBCT image of a 14‐year‐old female with condylar surface irregularity and erosion. (b) A sagittal CBCT image of a 15‐year‐old male with a typical osteophyte formation

PR (ProMax; Planmeca, Helsinki, Finland) and CBCT (Alphard VEGA; Asahi Roentgen, Kyoto, Japan) were performed for all patients. PR and CBCT images were independently and subjectively evaluated by two experts who were blinded to patient clinical information that would bias interpretation of alterations in the TMJ. PR (Figure 1a) and CBCT (Figure 1b,c) images were analyzed dichotomously by the experts and defined as either “no evidence of OA” (non‐OA, normal) or “TMJ–OA.” A normal condyle was defined as round or oval in shape in the axial plane and convex, round, or flat in the coronal plane (Yale, Allison, & Hauptfuehrer, 1966). In the sagittal plane, the condyle should be round, and S shaped, with an intact smooth cortical outline. Change in the appearance of the TMJ, indicating TMJ–OA, was determined if there was sclerosis, erosion, deformity, flattening, or osteophyte formation (Brooks et al., 1997; Figure 2a,b).

### 2.3 Bone scintigraphy

#### 2.3.1 Image acquisition

Bone scintigraphy was performed on 712 TMJs in 356 symptomatic patients. An intravenous dose of ^99m^Tc‐hydroxymethylene diphosphonate, determined by 740 MBq × body weight/70 kg, was injected intravenously (Stauss, Hahn, Mann, & De Palma, 2010). After 3 hr, images were acquired using dual‐head gamma‐cameras (ECAM, Siemens, Munich, Germany) equipped with a low‐energy, high‐resolution collimator. Whole body frontal and lateral views of the right and left regions of the TMJ and cranium were obtained.

#### 2.3.2 Qualitative analysis

**Figure 3:**
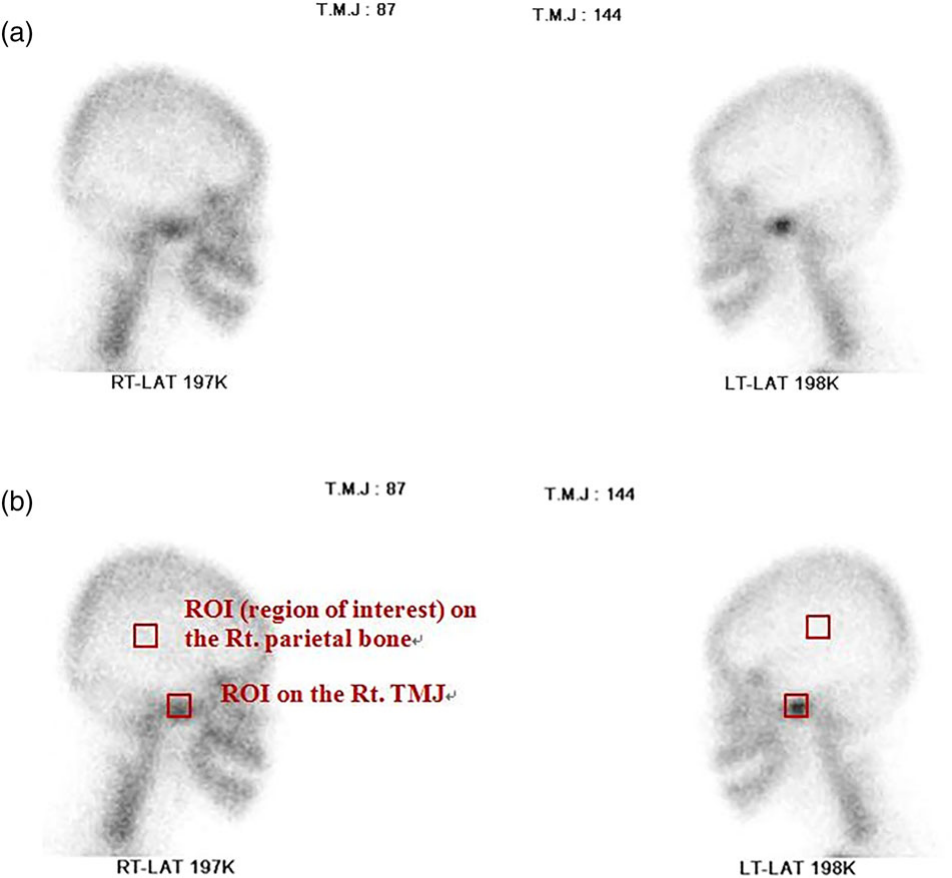
Bone scintigraphy. (a) Bone scintigraphy image showing increased uptake of the left condyle (qualitative analysis) and (b) quantitative analysis

For qualitative analysis, two board‐certified nuclear medicine radiologists assessed bone scintigraphy images subjectively and visually. The simple uptake level of ^99m^Tc‐hydroxymethylene diphosphonate by the TMJ against the ipsilateral/contralateral parietal bone and contralateral TMJ was evaluated for the detection of OA. The simple uptake of the TMJ was considered positive for TMJ–OA when it was higher than that of the contralateral TMJ or adjacent bone. When no abnormal or increased uptake was observed in either TMJ, the results were considered negative for TMJ–OA (Figure 3a). When a disagreement arose regarding the presence or absence of TMJ–OA in the reading results, consensus was achieved through discussion.

#### 2.3.3 Quantitative analysis

For quantitative analysis, a square region of the area of interest (13 × 13 pixels) was designated, and the counts in both the TMJ and parietal bones were measured (Figure 3b). The uptake ratio was calculated using the following formula. The simple uptake of the parietal bone was used as a background measurement for the TMJ regions.

$$\text{Uptake ratio}\;=\;\left(\mathrm{TMJ}\;\text{uptake}\;–\;\text{parietal uptake}\right)/\text{parietal uptake}.$$

### 2.4 Statistical analysis

Descriptive analyses were performed to determine the frequency and percentage of the analyzed variables. The Kruskal–Wallis test revealed that our parameters were nonnormally distributed. The chi‐squared test was used to analyze differences in categorical variables among the groups, such as sex and the presence of a chief complaint, whereas the Mann–Whitney *U* test was used with Tukey's post hoc analysis for continuous variables.

Intraimaging modality reliability were estimated using the kappa (κ) statistic: Cohen's kappa was calculated to determine the level of agreement regarding the presence of TMJ–OA in the dichotomized images (Cicchetti, 2001). Cramer's V test was also used to measure the strength of the association between the results of the diagnostic modalities.

Additionally, validity was measured as sensitivity and specificity (Parikh, Mathai, Parikh, Chandra Sekhar, & Thomas, 2008). The sensitivity, specificity, and the 95% confidence intervals of bone scintigraphy were calculated with CBCT and clinical assessment as the gold standards. The positive predictive value (PPV), negative predictive value (NPV), and error rate were also calculated. By analyzing the receiver operating characteristic (ROC) curve, the optimal cutoff values for the diagnosis of TMJ–OA from bone scintigraphy parameters and uptake ratios were obtained.

The data were analyzed using IBM's Statistical Package for the Social Sciences version 20.0 (SPSS Inc., Chicago, IL). A *P* value < 0.05 was considered statistically significant.

## 3 RESULTS

### 3.1 Clinical findings

Table 1 shows the distribution of demographics and the clinical findings of the three groups. The male to female ratio did not differ among the groups; females were predominant in all age groups. Temporomandibular dysfunction indices including the dysfunction index and craniomandibular index were significantly higher in young adults (Group 3) than in the early‐mid adolescents (Group 1). In addition, the visual analog scale pain intensity score of Group 3 was significantly higher than that of Groups 1 and 2. The most common chief complaint was TMJ noise (*n* = 293, 82.3%), followed by TMJ pain (*n* = 282, 79.2%) and mouth opening limitation (*n* = 32, 9.0%). The presence of mouth opening limitation in Group 1 was significantly lower than that in the older age groups.

**Table 1:** Demographic and clinical findings according to the group

Variable	Group 1	Group 2	Group 3	*P* value	Post hoc analysis
	Aged 12–16 (*n* = 115)	Aged 17–19 (*n* = 167)	Aged 20 (*n* = 74)		
Sex distribution					
Male, *n* (group%)	53 (46.1)	67 (40.1)	23 (31.1)	0.121	
Female, *n* (group%)	62 (53.9)	100 (59.9)	51 (68.9)		
Age (mean ± *SD*)	14.69 ± 1.04	18.05 ± 0.79	20.32 ± 0.71	**<0.0001** ***	**1–2**, **1–3**, **2–3**
Symptom duration	307.9 ± 398.7	485.3 ± 625.5	549.9 ± 715.3	**0.009** **	**1–2**, **1–3**
Pain intensity					
VAS (mean ± *SD*)	4.10 ± 2.34	4.19 ± 2.42	5.12 ± 2.53	**0.006** **	**1–3**, **2–3**
TMD indexes					
PI (mean ± *SD*)	0.08 ± 0.10	0.08 ± 0.11	0.09 ± 0.10	0.953	
DI (mean ± *SD*)	0.39 ± 0.19	0.42 ± 0.18	0.46 ± 0.18	**0.005** **	**1–3**
CMI (mean ± *SD*)	0.24 ± 0.13	0.25 ± 0.13	0.27 ± 0.11	**0.021** *	**1–3**
Chief complaints					
Reported‐TMJ pain, *n* (group%)					
No	32 (27.8)	29 (17.4)	13 (17.6)	0.078	
Yes	83 (72.2)	138 (82.6)	61 (82.4)		
TMJ noise, *n* (group%)					
No	23 (20.0)	27 (16.2)	13 (17.6)	0.709	
Yes	92 (80.0)	140 (83.8)	61 (82.4)		
Mouth opening limitation, *n* (group%)					
No	114 (99.1)	143 (85.6)	67 (90.5)	**<0.0001** ***	**1–2**, **1–3**
Yes	1 (0.9)	24 (14.4)	7 (9.5)		

*Note*. VAS: visual analog scale; PI: palpation index; DI: dysfunction index; CMI: craniomandibular index. Group 1: early‐mid adolescents aged 12 and16 years, Group 2: late adolescents aged 17 and19 years, Group 3: early adults aged 20 years. 1–2: When the significant difference existed between Groups 1 and 2. 1–3: When the significant difference existed between Groups 1 and 3. 2–3: When the significant difference existed between Groups 1 and 3. Results were obtained via Kruskal–Wallis test, Mann–Whitney *U* test, and chi‐squared test. *P* value significance was set at <0.05. Significant variables showed in bold text.

* *P* < 0.05.

** *P* < 0.01.

*** *P* < 0.001.

### 3.2 Distribution of diagnostic agreement between imaging modalities

Table 2 shows the distribution of diagnostic agreement between imaging modalities. To examine the TMJ–OA concordance rate among the imaging techniques, statistical analysis was performed. The concurrence of the results obtained from CBCT and PR was fair (κ value: 0.337–0.490), with the highest agreement observed in Group 3. However, the agreement rate between bone scintigraphy and the other imaging techniques, including CBCT and PR, was considerably low (κ value: 0.002–0.046). These low values can be interpreted as indicating that bone scintigraphy and other imaging techniques based on X‐ray obtain completely different results with respect to TMJ–OA diagnosis.

**Table 2:** The kappa values for the TMJ–OA diagnosis between imaging modalities

Group		Normal	TMJ–OA	κ value	*P* value
Group 1		CBCT			
BS	Normal	1	0	0.010	0.446
	TMJ–OA	72	42		
		PR			
BS	Normal	1	0	0.002	0.732
	TMJ–OA	102	12		
		PR			
CBCT	Normal	73	0	**0.337**	**<0.0001** ***
	TMJ–OA	30	12		
Group 2		CBCT			
BS	Normal	9	2	0.046	0.144
	TMJ–OA	93	63		
		PR			
BS	Normal	10	1	0.010	0.572
	TMJ–OA	132	24		
		PR			
CBCT	Normal	102	0	**0.433**	**0.0001** **
	TMJ–OA	40	25		
Group 3		CBCT			
BS	Normal	2	1	0.019	0.655
	TMJ–OA	38	33		
		PR			
BS	Normal	3	0	0.023	0.353
	TMJ–OA	55	16		
		PR			
CBCT	Normal	40	0	**0.490**	**<0.0001** ***
	TMJ–OA	18	16		

*Note*. PR: panoramic radiography; CBCT: cone beam computed tomography; BS: bone scintigraphy; OA: osteoarthritis on TMJ. TMJ: temporomandibular joint. Results were obtained via kappa statistics. *P* value significance was set at <0.05. Significant variables showed in bold text.

** *P* < 0.01.

*** *P* < 0.001.

### 3.3 Distribution of TMJ–OA diagnoses according to imaging modalities

Table 3 shows the distribution of TMJ–OA diagnoses according to imaging modalities. Overall, the diagnosis of TMJ–OA using PR (*n* = 53, 14.5%) exhibited the lowest value compared with the other imaging modalities. The diagnosis rate of TMJ–OA based on CBCT was 39.6% (*n* = 141). The overall presence of reported TMJ pain (*n* = 282, 77.3%) was closest to the results of bone scintigraphy (*n* = 333, 91.2%), which was markedly high. Because bone scintigraphy had a considerably high rate of TMJ–OA, which was higher than the rate of reported TMJ pain, careful interpretation may be necessary. The rate of TMJ–OA diagnosis using PR (16 of 74, 21.6%) and CBCT (34 of 74, 19.7%) was the highest in Group 3, the oldest group. Conversely, the value of bone scintigraphy was highest in Group 1 (114 of 115, 99.1%), followed by Group 2 (155 of 167, 92.8%) and Group 3 (61 of 74, 82.4%). In Group 2, the diagnostic rate of TMJ–OA was higher in females than in males for all three imaging modalities, but the difference was statistically significant only on PR and CBCT but not on bone scintigraphy.

**Table 3:** Comparison of the presence rate of TMJ–OA between diagnostic modalities

		Group 1		*P* value	Group 2		*P* value	Group 3		*P* value
		Male, *n*=53, (%)	Female, *n*=62, (%)		Male, *n*=67, (%)	Female, *n*=100, (%)		Male, *n*=23, (%)	Female, *n*=51, (%)	
PR	Normal	50 (94.3)	53 (85.5)	0.122	63 (94.0)	79 (79.0)	**0.008** **	19 (82.6)	39 (76.5)	0.762
	OA	3 (5.7)	9 (14.5)		**4 (6.0)**	**21 (21.0)**		4 (17.4)	12 (23.5)	
CBCT	Normal	32 (60.4)	41 (66.1)	0.523	49 (73.1)	53 (53.0)	**0.009** **	12 (52.2)	28 (54.9)	0.828
	OA	21 (39.6)	21 (33.9)		**18 (26.9)**	**47 (47.0)**		11 (47.8)	23 (45.1)	
BS^a^, ^b^	Normal	0 (0.0)	1 (1.6)	1.000	5 (7.5)	7 (7.0)	1.000	6 (26.1)	4 (7.8)	0.061
	OA	53 (100.0)	61 (98.4)		62 (92.5)	93 (93.0)		17 (73.9)	47 (92.2)	
OA by reported‐TMJ pain	Normal	13 (24.5)	19 (30.6)	0.466	18 (26.9)	11 (11.0)	**0.008** **	3 (13.0)	10 (19.6)	0.743
	OA	40 (75.5)	43 (69.4)		**49 (73.1)**	**89 (89.0)**		20 (87.0)	41 (80.4)	

*Note*. PR: panoramic radiography; CBCT: cone beam computed tomography; BS: bone scintigraphy; OA: osteoarthritis on TMJ. Group 1: early‐mid adolescents aged 12 to16years; Group 2: late adolescents aged 17 to19years; Group 3: early adults aged 20years. Results were obtained via Fisher's exact test. *P* value significance was set at <0.05. Significant variables showed in bold text.

a When the distribution of TMJ–OA is significantly different between Groups 1 and 2.

b When the distribution of TMJ–OA is significantly different between Groups 1 and 3.

** *P*<0.01.

### 3.4 The relationships between reported TMJ pain and TMJ–OA

Table 4 shows associations between the TMJ–OA diagnosed using each technique and the TMJ pain reported by the patient. Cramer's V test was used to measure the strength of the association between two nominal variables, including the results (the presence of TMJ–OA) of each modality and the presence of reported TMJ pain. Consequently, reported TMJ pain was significantly associated only with the results of bone scintigraphy and not with the results of PR or CBCT.

**Table 4:** The relationships between reported TMJ pain and TMJ–OA

	Reported TMJ pain	
	Cramer's V	*P* value
Group 1		
PR	0.148	0.113
CBCT	0.053	0.574
BS	**0.428** **	**<0.0001** ***
Group 2		
PR	0.029	0.708
CBCT	0.009	0.905
BS	**0.329** **	**<0.0001** ***
Group 3		
PR	0.156	0.184
CBCT	0.002	0.987
BS	**0.372** **	**0.001** **

*Note*. PR: panoramic radiography; CBCT: cone beam computed tomography; BS: bone scintigraphy; TMJ: temporomandibular joint. Results were obtained via Cramer's V test. *P* value significance was set at <0.05. Significant variables showed in bold text.

** *P* < 0.01.

*** *P* < 0.001.

### 3.5 Cutoff value for TMJ–OA

The sensitivity, specificity, PPV, NPV, and error rate of bone scintigraphy for TMJ–OA are shown in Table 5. For TMJ–OA, bone scintigraphy and CBCT showed higher sensitivity than PR, but no statistical significance was achieved, given the small number of TMJ–OA diagnoses. The sensitivity and specificity when the cutoff value of the uptake ratio was 2.0 ranged from 68.9% to 80.7% and from 33.3% to 46.4%, respectively. The overall sensitivity was higher than the specificity in all age groups. Sensitivity was highest in Group 3, whereas specificity was highest in Group 2. Although the specificity was relatively low, it can be concluded that bone scintigraphy is a useful imaging modality to screen for TMJ–OA because of its high sensitivity.

**Table 5:** Sensitivity, specificity, PPV, NPV, and error rate of bone scintigraphy

Cutoff value with uptake ratio of 2.0		Bone scintigraphy		Sensitivity (%) [95% CI]	Specificity (%) (95% CI)	PPV (%) [95% CI]	NPV (%) [95% CI]	Error rate (%)	*P* value
		Normal	OA						
Group 1	Normal (<2.0)	4	32	68.9	33.3	89.9	11.1	34.8	**<0.05** *
	TMJ–OA (≥2.0)	8	71	[59.1, 77.7]	[9.9, 65.1]	[85.4, 93.1]	[5.1, 22.6]		
Group 2	Normal (<2.0)	13	36	74.1	46.4	87.3	26.5	30.5	**<0.01** **
	TMJ–OA (≥2.0)	15	103	[66.0, 81.2]	[27.5, 66.1]	[82.8, 90.8]	[18.2, 37.0]		
Group 3	Normal (<2.0)	4	12	80.7	33.3	86.2	25.0	27.0	**<0.01** **
	TMJ–OA (≥2.0)	8	50	[68.6, 89.6]	[9.9, 65.1]	[80.5, 90.5]	[11.4, 46.2]		

*Note*. OA: osteoarthritis on TMJ; CI: confidence interval; PPV: positive predictive value; NPV: negative predictive value; TP: true positive; TN: true negative; FP: false positive; FN: false negative. Sensitivity was obtained from TP/(TP + FN) × 100; specificity was obtained from TN/(TN + FP) × 100; PPV was obtained from TP/(TP + FP) × 100; NPV was obtained from TN/(TN + FN) × 100; error rate was obtained from (FN + FP)/(TN + TP + FN + FP) × 100. *P* value significance was set at <0.05. Significant variables showed in bold text.

* *P* < 0.05.

** *P* < 0.01.

### 3.6 ROC curve

**Figure 4:**
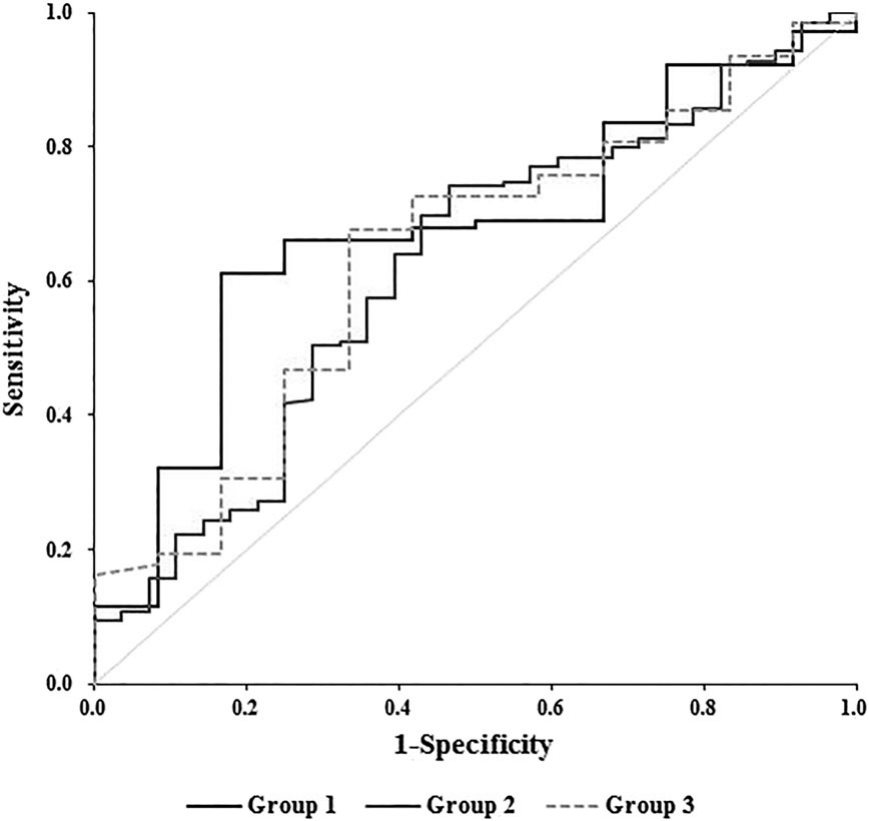
Receiver operating characteristic curve of the uptake ratio

The summary area under the ROC curve was investigated for evaluation of the effectiveness of bone scintigraphy procedures for the diagnosis of TMJ–OA (Figure 4). The present study aimed to assess the clinical validity and reliability of bone scintigraphy for the diagnosis of TMJ–OA, using interpretations of CBCT as the gold standard. The uptake ratio indicated that bone scintigraphy was effective in the results of Groups 1 and 2 (*P* < 0.05), and the area under the ROC curve ranged from 0.620 to 0.673, which were higher than the value of Group 3 (area under the ROC ranged from 0.607 to 0.613). Additionally, the optimal cutoff values of the uptake ratio for the diagnosis of TMJ–OA were 2.171 and 2.017 in Groups 1 and 2, respectively. When a diagnosis of TMJ–OA was made with a cutoff value for the uptake ratio of 2.0 or more, sensitivity was 68.93% and specificity was 60.0% (area under the curve = 0.73, *P* = 0.050). Thus, a cutoff value for the uptake ratio of 2.0 or more was used as a reference for diagnosing TMJ–OA in adolescents.

## 4 DISCUSSION

This study demonstrated the effectiveness and validity of bone scintigraphy in diagnosing TMJ–OA in adolescents by assessing the kappa value of intraimaging modality agreement, sensitivity, specificity, PPV, NPV, and the optimal cutoff value of the uptake ratio. Our results suggested that bone scintigraphy of the TMJ is a useful diagnostic method, matching the effectiveness of subjective TMD pain, and is more valuable in adolescents than in young adult patients with TMJ–OA and arthrogenic TMD. Signs and symptoms of TMD in children and adolescents have been reported since the early 1970s (Grosfeld & Czarnecka, 1977). Temporomandibular dysfunction pain is relatively common, occurring in approximately 10% of the population over the age of 18 (LeResche, 1997). No previous studies have been conducted on bone scintigraphy for the diagnosis of TMJ–OA in adolescents; however, our findings are in agreement with recent research on the effectiveness of bone scintigraphy in diagnosing TMJ–OA (Bag et al., 2014; Kim et al., 2012; Qing, Lei, Ma, Xie, & Deng, 2008).

Axis I of the RDC/TMD, a clinical and radiographic assessment tool, briefly describes the image analysis criteria for TMJ disc displacement using arthrography and magnetic resonance imaging (MRI) and TMJ–OA based on tomography (Dworkin & LeResche, 1992). Although bone scintigraphy and PR were not included as imaging options in the original RDC/TMD, they have been recommended as screening tools for TMJ pathology. It is well documented that clinical symptoms, such as pain, may not manifest even in the presence of severe erosive TMJ disease, and subjective symptoms may lead to an underestimation of the degree of early inflammation. In a previous study, the clinical signs of synovitis, including swelling, tenderness, and limited range of motion, facilitated accurate diagnosis of active TMJ inflammation in only 58% of patients (Hu, Schneiderman, & Harper, 1996). In another clinical trial, when no bone deformity was observed through conventional radiography, clinical diagnosis based on a detailed questionnaire, measurements of mouth opening and deviation, and evaluation of crepitus and pain achieved identification of only 39% of patients with active inflammation (Muller et al., 2009). Thus, no single modality meets every imaging requirement concurrently, and a combination of clinical diagnosis and imaging diagnosis is needed to the diagnosis of early TMD.

In our study, we used bone scintigraphy as a diagnostic imaging method, and it was more accurate than PR and CBCT in detecting TMJ–OA and self‐reported TMJ pain in adolescents. PR has frequently been used as a simple, low‐cost method to evaluate the bony structure of the TMJ. However, the depiction of the articular eminence and fossa is inadequate for diagnosis because of the nature of the imaging and superimposition of the base of the skull and zygomatic arch (Epstein et al., 2001). In contrast, CBCT is more accurate than PR for the high‐resolution assessment of bone components and morphological changes of the TMJ (Huntjens, Kiss, Wouters, & Carels, 2008) and widely used for the evaluation of TMJ. The use of bone scintigraphy in the diagnosis of TMD has been infrequent vcompared with these traditional radiographic techniques. Because of age‐dependent metabolism in the developing skeleton, the interpretation of bone scintigraphy in adolescents is challenging.

Nevertheless, bone scintigraphy has the potential to detect active bone destruction or remodeling, whereas corresponding radiographs may be normal or document past structural change in the joint (Epstein et al., 2002). As with other joints, radiography can demonstrate chronic erosion of the TMJ but is unable to help identify active synovial inflammation, osteitis, or joint effusion. Goldstein and Bloom (1980) also suggested that bone scintigraphy is a more useful tool than plain radiography or tomography for the diagnosis of degenerative changes of the TMJ. In our results, bone scintigraphy had relatively high sensitivity but low specificity in detecting adolescent TMJ–OA. To achieve more accurate and reliable results, future studies should investigate a larger group of adolescents with TMD with comparison against the results of a healthy control group without TMD.

In the present study, the proportion of patients with subjectively reported TMJ pain was most consistent with the rate of TMJ–OA diagnosed using bone scintigraphy, which may represent early TMJ–OA findings that other imaging modalities could not detect. Bone scintigraphy is useful for detecting the early stage of inflammatory arthritis (Qing et al., 2008). Detecting these destructive signs early in the course of the disease, both clinically and radiographically, is critical. According to a prospective cohort study, although there was no difference in TMJ‐related symptoms at baseline, after 15 years, children with juvenile arthritis reported a significantly higher prevalence of tiredness of the jaw, pain in the face or jaw, and difficulty in mouth opening (Engstrom et al., 2007). Furthermore, an early diagnosis should be made before extensive growth abnormalities are evident (Huntjens et al., 2008; Twilt, Mobers, Arends, ten Cate, & van Suijlekom‐Smit, 2004). The TMJ is susceptible to damage from arthritis because of its unique anatomy and biochemical composition.

In juvenile patients, this damage from TMJ–OA has been associated with several clinically important outcomes. It includes decreased chewing ability, midline deviation, convex facial profile, Class II malocclusion, crowded lower anterior teeth, anterior open bite, and reduction in the maximum opening of the mouth. That is, mandibular growth occurs within this center from the prenatal period until just after puberty, and damage to the growth center caused by inflammation or trauma during this time frequently results in alterations in mandibular growth (Ronchezel et al., 1995). Unlike other synovial joints, the mandibular growth center is located under a thin layer of fibrocartilage located at the surface of the condylar head. When articular cartilage is in a low‐oxygen environment, the chondrocyte response to hypoxic conditions can lead to further destructive changes (Mino‐Oka et al., 2017). Furthermore, even apparently minor radiographic changes have been associated with disrupted mandibular growth and major craniofacial changes (Billiau, Hu, Verdonck, Carels, & Wouters, 2007). Therefore, one of our aims was to highlight the importance of recognizing and treating TMJ–OA in the early stage, using bone scintigraphy. Early detection of juvenile TMJ–OA is critical to prevent long‐term complications, and additional bone scintigraphy imaging of the TMJ in adolescent patients with TMJ–OA or TMD symptoms is necessary.

Our study had a few limitations regarding the collection of the TMD sample. Our sample consisted of TMD patients who were aged 12–20 years. Although we excluded cases of oligoarthritis and polyarthritis through screening with bone scintigraphy, they might have TMJ involvement in juvenile idiopathic arthritis. The duration between symptom onset and diagnosis could be a factor in the presence of OA and the radiologic features observed. In our data, the symptom duration was significantly shorter in Group 1 than in Groups 2 and 3. However, a shorter period for younger adolescents to present at a hospital can be considered a distinct feature of younger adolescent TMD. In addition, standard MRI and more advanced techniques such as single‐photon emission computed tomography have improved the assessment of joint disease (Mohl, 1993). To establish the diagnostic reliability and validity of bone scintigraphy in TMJ–OA more clearly, comparison with MRI or other advanced imaging techniques may be necessary. Consequently, combining the results of bone scintigraphy with those of other imaging modalities and clinical observations can obtain a more accurate diagnosis of TMJ–OA.

## 5 CONCLUSION

Juvenile TMJ–OA commonly affects adolescents and is a major cause of both short‐ and long‐term disabilities; excessive delay in the prompt treatment of juvenile TMJ–OA can result in irretrievable damage to joints and impair skeletal maturation. Our results showed that bone scintigraphy for TMJ–OA is a complementary exam, and it may be one of the methods of choice for TMJ–OA diagnosis. Therefore, additional studies are necessary to improve the accuracy of the diagnosis of TMJ–OA using additional bone scintigraphy in adolescents.

## CONFLICTS OF INTEREST

The authors declare no conflicts of interest.

## ETHICAL APPROVAL

The present study was performed in accordance with the Declaration of Helsinki and was approved by the institutional review board of our institute.

## INFORMED CONSENT

Informed consent was obtained from all individual patients included in this study.
